# Curative Anti-Inflammatory Properties of Chinese Optimized Yinxieling Formula in Models of Parkinson's Disease

**DOI:** 10.1155/2018/6142065

**Published:** 2018-11-04

**Authors:** RenRong Wei, Jing OuYang, WeiXian Lin, TongXiang Lin

**Affiliations:** ^1^Second Clinical Medical College, Guangzhou University of Chinese Medicine, 232 Waihuan Road E, Guangzhou, Guangdong 510006, China; ^2^Center for Regenerative and Translational Medicine, Guangdong Provincial Academy of Chinese Medical Sciences & Guangdong Provincial Hospital of Chinese Medicine, 111 Dade Road, Guangzhou, Guangdong 510120, China; ^3^Bee Science College, Fujian Agriculture and Forestry University, 15 Shangxiadian Rd., Fuzhou, Fujian 350002, China

## Abstract

Parkinson's disease (PD) is marked by the progressive degeneration of dopaminergic neurons (DAN) accompanied by glial activation. Thus, inhibiting glial activation that occurs during this disease could be an effective method for treating PD. Optimized Yinxieling Formula (OYF), a Chinese medicinal formula, which is used to efficiently treat autoimmune disease psoriasis, has been proved to display potential immunomodulatory effects in inflammation-associated diseases. This study assessed the therapeutic benefits of OYF on glial-mediated neuroinflammation and neuroprotection in PD models* in vitro* and* in vivo. *First, the results showed that OYF significantly suppresses LPS-induced proinflammatory cytokine secretion and attenuates the overall inflammatory responses in BV-2 cells. Second,* in vivo* studies confirm that while the validity of our MPTP-induced PD mouse models possesses activated glia and significant neurobehavioral dysfunction, pretreatment with OYF prevents glial activation and ameliorates movement dysfunction in the MPTP-induced PD mouse models as evaluated by the pole and rotarod tests. Third, transcriptomic analyses were carried out to reveal the underlying molecular mechanism of the OYF treatment. Sixteen pathways were significantly upregulated in the OYF-treated PD model mice, including the cytokine-cytokine receptor interaction, cell adhesion molecules, coagulation, and complement cascades. Fifteen pathways were significantly downregulated in the OYF-treated PD model mice, such as the natural killer cell mediated cytotoxicity, hematopoietic cell lineage, phagosome, and others. These pathways share direct or indirect features of immunomodulation, suggesting that the physiological effects of OYF involve key roles of immune and inflammation regulations. Therefore, we prove that OYF is a useful immunomodulatory formula in developing prevention and treatment methods for neurodegenerative disease PD.

## 1. Introduction

Of all neurodegenerative disorders, Parkinson's disease (PD) is the second most frequently encountered neurodegenerative condition after Alzheimer's disease. The hallmark of PD is the progressive degeneration of DAN in the substantia nigra pars compacta (SNpc). The resultant striatal dopamine (DA) depletion leads to irreversible motor dysfunction such as resting tremor, rigidity, bradykinesia, and gait disturbances [1]. PD therapy is currently based on the replacement of the DA neurotransmitter, which can relieve clinical symptoms but cannot prevent death of DAN[2]. Therefore, it is necessary to develop new efficient candidates based on PD pathogenesis.

Several factors such as aging, environment, and heredity have been implicated in PD etiology. Nevertheless, the mechanisms culminating in PD neuronal loss has yet to be fully understood. In the past few decades, mounting evidence demonstrated that innate and adaptive immunity affected the pathogenesis of PD [3–7]. In PD, neuroinflammation is thought to be mediated primarily by microglial activation, the resident cerebral immune cells. Activated microglia can directly cause DAN damage through the overproduction of proinflammatory cytokines, such as NO, TNF-*α*, IL-1*β*, and IL-6. Moreover, the activation of microglia also leads t**o astrogliosis while **simultaneously attracting an influx of peripheral immune cells (a majority of which are T cells), and together creating a vicious network of inflammation [8–10]. New evidence in recent years points to PD being a type of autoimmune disease [11–14]. Taken together, immune responses have been firmly established in its contribution towards the pathogenesis of PD and may be a potential target in the development of drugs to prevent DAN death.

In traditional Chinese medicine (TCM), PD is termed as “shaking palsy”. Clinically, to treat PD in China, Chinese herbal medicines are prescribed either alone or as a formulation created based on TCM theory or modern pharmacological theories for oral administration. A fair number of TCM formulae have been investigated in animal experiments and clinical trials [15–19].

OYF was optimized from an internal-hospital preparation of the Yinxieling tablet, which was developed by Guowei Xuan, a Chinese medicinal master in Guangdong Provincial Hospital of Chinese Medicine [20]. OYF, a concoction of* Curcuma zedoaria*,* Glycyrrhiza uralensis, dark plum fruit, *and other 4 herbal medicines, has successfully performed in treating moderate and severe psoriasis vulgaris without significant side effects [21]. The therapeutic action of OYF in psoriasis is thought to cause inhibition of keratinocyte proliferation through the downregulation of cyclin B2 [22] or the inhibition keratinocyte release of inflammatory cytokines and chemokines via NF-*κ*B signaling pathway downregulation [23]. Based on the immunoregulatory effects and potential neuroprotective effects of OYF, this study aimed to further investigate the therapeutic effects of OYF in PD through PD rodent models.

## 2. Materials and Methods 

### 2.1. Reagents

1-Methyl-4-phenyl-1,2,3,6-tetrahydropyridine (MPTP) and LPS were bought from Sigma Aldrich (St. Louis, MO, USA). 3-(4,5-Dimethylthiazol-2-yl)-5-(3-carboxymethoxyphenyl)-2-(4-sulfonatophenyl)-2H-tetrazol-3-ium (MTS) was obtained from Promega (Madison, WI, USA). UltraVision LP Detection System HRP Polymer and DAB Plus Chromogen were obtained from Thermo Fisher Scientific (Fremont, CA, USA). Fetal bovine serum (FBS) and Dulbecco's modified Eagle's medium (DMEM) were procured from Gibco-BRL Technologies (Carlsbad, CA, USA). Culture plates were from Nunc Inc. (Aurora, IL, USA).

### 2.2. OYF Formula

OYF is a concoction of Curcuma zedoaria, Glycyrrhiza uralensis, dark plum fruit, Lithospermum erythrorhizon, Paeonia lactiflora, Sarcandra glabra, and Rhizoma Smilacis Glabrae and at a ratio of 3:2:5:2:3:5:5. OYF granules were prepared in our hospital (Guangdong Provincial Hospital of Chinese Medicine).

Before application, the OYF granules were identified and qualified with liquid chromatography coupled with an LTQ Orbitrap MS refereed to previous studies [24]. 3-O-Caffeoylquinic acid, paeoniflorin, liquiritin, astilbin, engeletin, liquiritigenin, and glycyrrhizic acid were the main active components of OYF.

### 2.3. Preparation of OYF Containing Serum (OYFCS)

Adult male SD rats, body weight 250±20g, were bought from the Beijing Vital River Laboratory Animal Technology Co., Ltd. (Beijing, China). All animal experimental protocols were approved and performed in accordance with our Institutional Animal Care Guidance.

Thirty rodents were randomly assigned into an OYF group or a control group. OYF doses administered to rats (6.45 g/kg) were 5 times the daily adult dosage. Rats of the OYF group were given medicine by gastric perfusion while rats of the control group were given the same volumes of saline solution. All rats were sacrificed under pelltobarbitalum natricum anesthesia after 3 days of administration via exsanguination from the abdominal aorta. Blood was collected in sterile test tubes which were subjected to centrifugation at 3000 r/min for 15 minutes. For deactivate complements, the resultant serum was extracted and heated at 56°C for 30 minutes and was finally filtered through a 0.22*μ*m filter for bacteria removal and maintained at −20°C.

### 2.4. MPTP-Induced PD Mouse Model

Eight-week-old male adult C57BL/6 mice (25±3g) were bought from the Beijing Vital River Laboratory Animal Technology Co., Ltd. (Beijing, China) and acclimatized in standard cages under controlled temperature (23 ± 3°C) and a regular 12h:12h light-dark cycle for 7 days before the procedure. All mice were divided randomly into the following groups: normal control group (n=12; 0.9% saline, intraperitoneal (i.p.)), MPTP group (n=12; 18 mg/kg MPTP in 0.9% saline, 4 times of injections carrying on for every 2 hours in a day, i.p.), and MPTP + OYF group (n=12; 18 mg/kg MPTP given via four i.p injections/day for every 2-hour intervals + 2.58 g/kg OYF orally (p.o.)). Mice were treated with 2.58 g/kg of OYF p.o. for 7 days prior to MPTP injection.

### 2.5. Motor Activity Tests

#### 2.5.1. Pole Test

A mouse was placed at the top-most end of a vertical 60cm tall rough-surfaced pole that was 1cm in diameter. The time taken for the mouse to reach the floor was considered the duration of time for the pole test. A mouse was determined to suffer from bradykinesia should it take longer than usual to climb down the pole. Each mouse was subjected to this test 5 times in succession.

#### 2.5.2. Rotarod Test

A Rotarod test (Ugo 47600, Italy) which consisted of five rotating rods of 3cm in diameter was used to evaluate mice motor coordination. Prior to the actual test, mice were allowed a few trials on the instrument for familiarization. For the actual test, mice were placed on a rotating rod that took 180 seconds to accelerate from 5 to 20 rpm. The duration of time that each mouse was able to stay on the rotating rod was recorded, and mice that were successful in finishing the test were allocated a latency time of 180 seconds.

### 2.6. Cell Culture and Treatment

The BV-2 cells of murine origin were cultured in 10% FBS-supplemented DMEM with penicillin-streptomycin. Cells were seeded overnight onto plates. The next day, BV-2 cells were exposed to different concentrations of OYFCS (2.5, 5, and 10%) for 2 hours, followed by the addition of LPS (100ng/mL) or LPS alone for the indicated times (6, or 24h).

### 2.7. Immunohistochemistry

Immediately after anesthesia, mice were perfused transcardially with chilled 4% paraformaldehyde (PFA). Mouse brains were dissected, fixed in 4% PFA, and paraffin-embedded. A microtome (Leica, Germany) was used to section 3*μ*m-thick coronal sections. For immunohistochemical staining, sections were first dewaxed and hydrated before exposed to a pH 6.0 citric acid buffer in a microwave for antigen retrieval. Sections were then exposed to 3% hydrogen peroxide for 15 minutes to exhaust endogenous peroxidase activity, followed by three washes in phosphate buffered saline (PBS). After a 10-minute blocking with hydrogen peroxide at room temperature, the sections were incubated for 20 minutes with primary antibodies at room temperature. The following primary antibodies were used: Iba-1 1:2000, anti-TH 1:1000, and GFAP 1:1000. All antibodies were obtained from Abcam, Cambridge, UK, and were suspended in 5mL 0.1M PBS. Sections were then incubated for 10 minutes with primary antibody enhancer at room temperature, followed by another 15-minute incubation with HRP-conjugated secondary antibody at room temperature and lastly subjected to three washes with PBS. 3,3-Diaminobenzidine (DAB) was used for color development and was left on the sections for a maximum of 2 minutes. Finally, the sections were mounted on slides and dehydrated with an ethanol gradient.

An Olympus BX61 microscope was used to capture images. The ImageJ program was used to analyze target protein staining intensity in DAN, microglia, and astroglia cells in a double-blinded fashion. Four sections of the midbrain were chosen randomly, with a total of 12 sections per mouse based on unbiased stereological rules. Sample images depict analysis of the substantia nigra. The control group was used for data normalization.

### 2.8. Total RNA Extraction and Real-Time PCR

Total RNA extraction was carried out using the TRIZOL reagent (Invitrogen, USA) based on manufacturer's instructions. The purity of RNA samples was assessed with a NanoDrop, and samples with ratios between 1.8 and 2.01 were used for cDNA synthesis using the GoScript™ Reverse Transcription System (Promega, USA). The qPCR reaction was performed in 20 *μ*l with 5*μ*l template DNA and 500 nM primers.  Table 1 lists all primers used in these reactions. Each sample was analyzed in triplicate. The iTaq™ Universal SYBR® Green Supermix (Bio-Rad, USA) were supplemented under the guidance of manufacturer's instruction. The PCR results were analyzed in a Real-Time PCR system (Bio-Rad, CFX96, USA).

### 2.9. Transcriptome Analysis

The global gene expression patterns of brains from the untreated control, untreated PD model, and OYF-treated PD model were analyzed to determine differentially expressed genes (DEGs) in response to OYF treatment. Gene expression data were first normalized to 0, log2 (V1/V0) and log 2(V2/V0) and subjected to clustering via the Short Time-series Expression Miner software (stem) [25]. Data parameters were fixed at the following:

- Maximum change of units in model profiles between time points is 1.
- Maximum number of output profiles is 20, with similar profiles merged.
- Minimum DEGs fold change ratio of no less than 1.5 (2.0).
- Significant clustered profiles were those determined to have a P value < or equal to 0.05. The DEGs in all or each profile then underwent subsequent gene ontology (GO) and KEGG pathway enrichment analysis. GO terms or KEGG pathways that had p values of less than 0.05 were determined to be significantly enriched.

### 2.10. Statistical Analysis

The data was analyzed with the SPSS 21 software and expressed as the mean ± SD. Differences were analyzed using one-way ANOVA with post hoc LSD* t*-test or a Tamhane's T2 test. A P-value of less than 0.05 was taken to confer statistical significance.

## 3. Results

### 3.1. OYFCS Inhibits NO Production in LPS-Activated BV-2 Cells

Due to the fact that actual effective components in the mouse model are not the formula itself but the OYFCS, we applied the OYFCS to test the cells in vitro.

**Figure 1:**
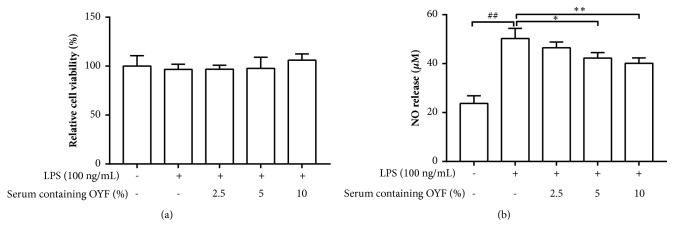
**The effect of OYFCS in LPS-stimulated BV-2 cells.** Twenty-four hours prior to the LPS treatment (100ng/mL), BV2 cells were subjected to a two-hour incubation with OYFCS at different concentrations. An MTS assay was used to evaluate cell viability. Results are given as a percentage of viable cells in comparison to control (a). Levels of NO were tested in culture supernatants using the Griess reaction (b). Values are depicted as mean ± standard deviation (n=3) for three independent experiments. ^##^*P*< 0.01 versus control group and ^*∗*^*P*< 0.05, ^*∗∗*^*P*< 0.01 versus LPS-treated one.

At first, cytotoxicity was tested in BV-2 cells supplemented with various concentrations of OYFCS (2.5, 5 and 10%) and/or LPS (100 ng/mL). MTS assay of the treatments revealed that no difference among LPS treatment, OYFCS, and control group is found, suggesting that both of OYFCS and LPS were safe for the cells at the prescribed concentrations (Figure 1(a)).

Secondly, to evaluate the ability of OYFCS to attenuate NO secretion by BV-2 cells upon LPS exposure, cells were incubated for 2 hours with 2.5, 5 and 10% of OYFCS before exposure to LPS (100 ng/mL). On one hand, LPS exposure demonstrated significant elevations in NO levels (50.3 ± 4.1 *μ*M, P< 0.001) in contrast to 23.7 ± 3.1 *μ*M in control cells. On the other hand, pretreatment of 2.5, 5, and 10% of OYFCS in LPS-treated cell cultures demonstrate a potential decrease in dosage dependence of NO level, with their respective NO levels being 46.5 ± 2.4, 42.3 ± 2.2, and 40.1 ± 2.2 (P< 0.01) *μ*M (Figure 1(b)). The NO level was significantly inhibited by the supplementation of the 10% OYFCS compared to LPS vehicle although the data was still much higher than those in the control.

### 3.2. Effect of OYFCS on TNF-*α*, IL-1*β*, and IL-6 Cytokine Expression in LPS-Stimulated BV-2 Cells

**Figure 2:**
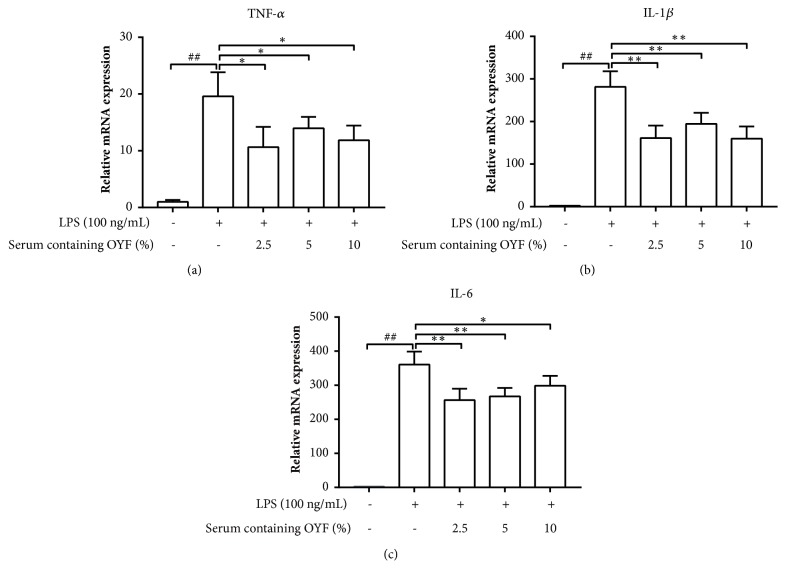
**Suppression of proinflammatory cytokines expression by OYFCS in LPS-stimulated BV-2 cells.** The mRNA expression levels of TNF-*α*, IL-1*β*, and IL-6 were measured by RT-PCR (a-c). *β*-actin was used as the internal control. Values are depicted as mean ± standard deviation (n=3) for three independent experiments.^##^*P*< 0.01 versus control group and ^*∗*^*P*< 0.05, ^*∗∗*^*P*< 0.01 versus LPS-treated group.

To determine if OYFCS is able to suppress LPS-triggered proinflammatory cytokine (TNF-*α*, IL-1*β*, and IL-6) production, BV-2 cells were treated with LPS (100 ng/mL) both with and without 2.5, 5, or 10% OYFCS. Six hours after exposure to LPS, RT-PCR analysis demonstrated the effects of the drug treatments. On the one hand, significant increases of proinflammatory cytokine mRNA expressions were observed in BV-2 microglia culture. On the other hand, this elevation was suppressed in BV-2 microglia cells that received OYFCS pretreatment for 2 hours (Figures 2(a)-2(c)). Taken together, this indicates that OYFCS works at a transcriptional level to halt the expressions of these cytokines (Figure 2 and Supplementary Figure 1).

### 3.3. Effect of OYF on Microglial and Glial Cell Activation in the Brains of the MPTP-Induced Mouse Models

**Figure 3:**
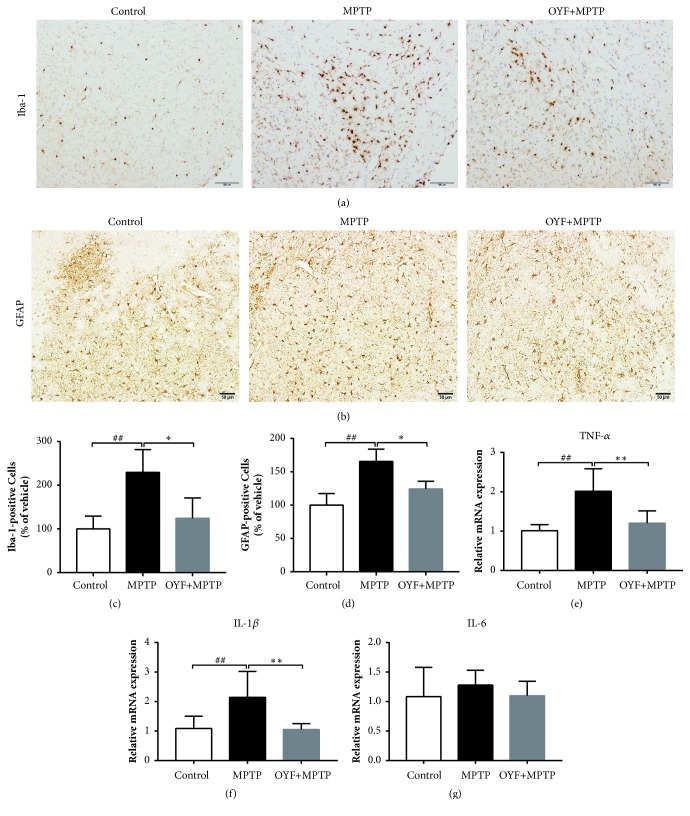
**The glial response in the PD mouse model treated with OYF.** The representative IHC staining for Iba-1^+^ microglia (a) and GFAP^+^ astrocytes (b) in SNpc. Scale bar = 100or 50 *μ*m. The quantitative percentage of area occupied by Iba-1^+^ cells (c) and GFAP^+^ cells (d) is shown in the indicated region of SNpc. (e-g) RT-PCR analysis of TNF-*α*, IL-1*β*, and IL-6 expression in mouse midbrain. For PCR, *β*-actin was used as an internal control. Data is depicted standard deviation. ^##^*P*< 0.01 versus control group; ^*∗*^*P*< 0.05 versus MPTP-treated group using one-way ANOVA with post hoc LSD* t*-test, n=6.

Rodents exposed to MPTP have been documented of displaying activation of cerebral microglia. As such, we utilized an MPTP-triggered PD mouse model in order to assess the protective effect of OYF on microglial and glial cell activation. Glial marker glial fibrillary acidic protein (GFAP) and microglial marker ionized calcium-binding adapter molecule 1 (Iba-1) were utilized to identify their respective cells through immunohistochemistry staining. The microglial marker Iba-1 was increased from 100.0 ± 29.5 in control to 229.5 ±52.1 in the model mice (P<0.01), and OYF pretreatment significantly inhibited elevation of the microglial marker to 124.4 ±46.4 (P<0.05) (Figures 3(a) and 3(c)). Meanwhile, the area occupied by GFAP+ cells 165.6± 18.3 (P< 0.01, Figures 3(b) and 3(d) and Supplementary Figure 3) in the SNpc of the model and OYF pretreatment lowered the data to 124.2 ± 11.5 (P< 0.05, Figures 3(b) and 3(d)) in comparison to the control.

Mounting evidence demonstrate that inflammation plays a key role in PD pathogenesis. Once activated, microglia express several proinflammatory mediators such as TNF-*α*, IL-1*β*, and IL-6, which are closely associated with various inflammation-related diseases. OYF-treated groups had significantly lower levels of TNF-*α* and IL-1*β* mRNAs compared to vehicle groups (Figures 3(e)-3(g)). Similarly, we also detected the protein activity performed with ELISA as showed in Supplementary Figure 2. These results indicated that OYF exerts its protective effects by inhibiting proinflammatory cytokines at the transcriptional levels.

### 3.4. Protective Effect of OYF against MPTP-Triggered Depletion of Tyrosine Hydroxylase (TH)

**Figure 4:**
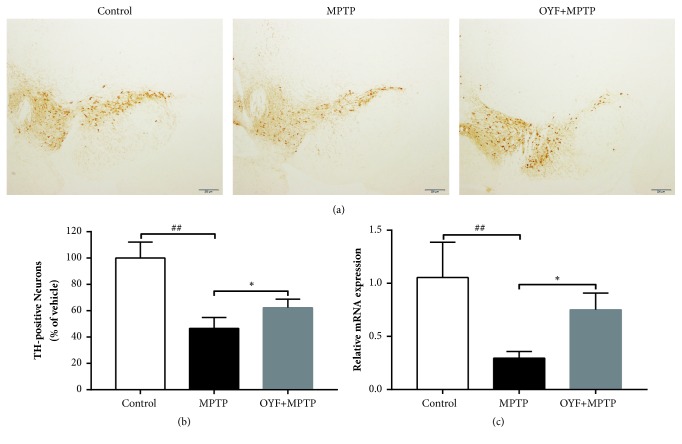
**The protective effects of OYF against MPTP-mediated neuronal damage in the SNpc.** Representative images of SNpc TH-positive cell immunoreactivity (IR) with scale bars, 200 *μ*m (n =6 per group) (a). Optical density (OD) tests for SNpc TH-positive cells (b). RT-PCR for TH mRNA expression in midbrain (c). For PCR, *β*-actin was used as an internal control. All the values are depicted as mean ± standard deviation. ^##^*P*< 0.01 versus control group and ^*∗*^*P*< 0.05 and ^*∗∗*^*P*< 0.01 versus MPTP-treated group using one-way ANOVA with post hoc LSD* t*-test, n=6.

Prior to receiving an intraperitoneal 18mg/kg MPTP injection, mice were first given per oral 2.58g/kg of OYF. There was a decrease in total TH-positive neurons after MPTP treatment alone (46.6 ± 8.3%, P< 0.01) in contrast to mice injected with saline only. On the other hand, mice that were also administrated OYF had intact numbers of cells with positive immunoreactivity for TH (percentage of immunoreactive cells: 62.2 ± 6.5%, P<0.05) (Figures 4(a) and 4(b) and Supplementary Figure 4 ). RT-PCR was also used to quantify TH mRNA expression (Figure 4(c)). The aforementioned findings allude towards the neuroprotective effect that OYF may confer against MPTP toxicity.

### 3.5. OYF Improves Performance in the Rotarod Test and the Pole Test in Mice Treated with MPTP

**Figure 5:**
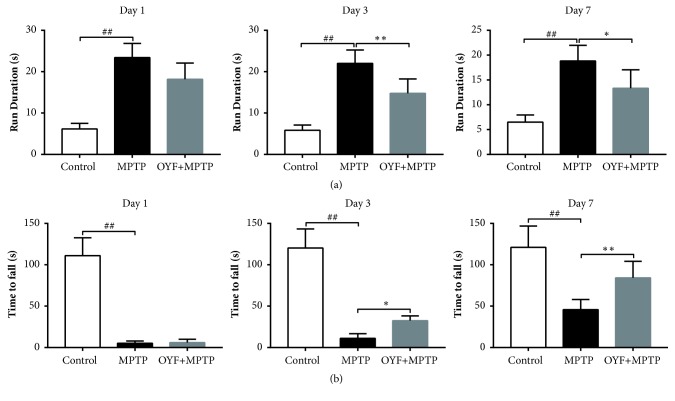
**The effects of OYF on the behavior in MPTP-intoxicated mice.** Pole test and rotarod test carried out on days 1, 3, and 7 following MPTP injection (18 mg/kg, 4 injection/day, 2-hour intervals,* i.p.*). Prior to MPTP injections, 2.58 g/kg*, p.o.* OYF was given for 7 consecutive days. Pole test (a) and rotarod test (b) were carried out. Values are depicted as mean ± standard deviation ((a-b) n = 12 per group). ^##^*P*< 0.01 versus control group and ^*∗*^*P*< 0.05 and ^*∗∗*^*P*< 0.01 versus MPTP-treated one.

Motor abilities in mice are critically affected after MPTP exposure, as evidenced by several motor-specific behavioral experiments. The duration of time taken to complete the pole test was markedly longer on day 1 (P< 0.01), day 3 (P< 0.01), and day 7 (P< 0.01) after exposure to MPTP. Pretreatment with OYF (2.58 g/kg) was able to significantly protect mice from increases in pole test times on day 3 (P< 0.01) and day 7 (P< 0.05) (Figure 5(a)). Mice were also subjected to rotarod tests, a measure of motor coordination. Mice that were given only MPTP suffered from shorter latent times on day 1 (P< 0.01), day 3 (P< 0.01), and day 7 (P< 0.01). Pretreatment with OYF (2.58 g/kg) was able to significantly protect mice from reducing the retention time on rotarod on day 3 (P< 0.05) and day 7 (P< 0.01) (Figure 5(b)). Surprisingly, we found that OYF appeared to ameliorate motor deficits induced by MPTP exposures in mice.

### 3.6. Transcriptomic Analyses Reveal the Underlying Molecular Mechanism of the OYF Treatment

A comprehensive transcriptomic analysis was performed to evaluate changes in gene expression after OYF treatment, followed by functional characterization of the DEGs by GO and KEGG. Two major trends were seen in the transcriptomic differences between the control, untreated model, and OYF-treated groups (summarized in Tables 2 and 3): profile 2 with control at normal level, untreated model at lower level and OYF-treated group restored to normal level, and profile 5 with control at normal level, untreated model at higher level and OYF-treated group restored to normal level.

In profile 2, 16 pathways were significantly upregulated in the OYF-treated mice, including the cytokine-cytokine receptor interaction, cell adhesion molecules (CAMs), coagulation and complement cascades, Jak-STAT signaling pathway, and leukocyte transendothelial migration et al. (*P*<0.05). In profile 5, 15 pathways were significantly downregulated in the OYF-treated mice, such as the natural killer cell mediated cytotoxicity, cell adhesion molecules (CAMs), coagulation and complement cascades, cytokine-cytokine receptor interaction, hematopoietic cell lineage, and phagosome (*P*<0.05) pathways. These pathways share the common feature of being immunomodulatory; although not all are directly involved in immunological responses, they are indirectly involved in regulating inflammatory responses. Based on the transcriptomic data, the physiological effect of OYF involves the regulation of several gene levels, especially immune and inflammation regulation.

## 4. Discussion

Current PD therapy is based on the replacement of the DA neurotransmitter, which can reduce clinical symptoms but cannot prevent DA neuronal loss, which is a fundamental problem in PD pathology. Therefore, it is necessary to develop a novel strategy and more efficient therapeutic candidates that can treat the root of PD pathogenesis.

In the past few decades, mounting evidence has demonstrated that the innate and adaptive immunity affects the pathogenesis of PD. Recent landmark discoveries suggest that PD may have an autoimmune component [11–14]. Given the significant role immune system plays in PD, targeting immune responses might serve as an effective therapeutic approach for PD, a newly discovered autoimmune disease.

The Chinese formula OYF has been reported to be an extremely efficient formula in treating psoriasis vulgaris, an autoimmune disease [26, 27]. Prior literature suggests that OYF may exert its therapeutic actions through the inhibition of inflammatory cytokine and chemokine release [23]. Moreover, we have found that OYF prevents excessive activation of the immune system in multiple sclerosis and in experimental autoimmune encephalomyelitis (EAE) mouse model of multiple sclerosis (unpublished data), both of which demonstrate features of autoimmunity. Based on the obtained data in these series of experiments, we hypothesized that OYF might be a potential therapeutic formula for PD in the rodent model.

We tested the therapeutic effects of OYF in a mouse PD model treated with MPTP. Our data showed that peritoneal treatment of MPTP to mice leads to typical symptoms of PD and obvious reduction of TH expression inSNpc, which is consistent with previous studies [28, 29]. In the behavior test, the mice pretreated with OYF (OYF+ MPTP) exhibited better grip strength and muscular coordination in the rotarod test and also obvious increased climbing ability with shorter time for climbing pole in comparison with those data in mouse PD model with MPTP only treatment. TH, a typical marker of DAN, catalyzes the rate-limiting step of hydroxylation of L-tyrosine to levodopa and is a reliable marker of the degree of PD development. In this study, while MPTP treatment imposed a significant reduction in TH-immunoreactivity, OYF treatment (MPTP+ OYF) significantly protected the dopaminergic (TH-positive neuron and TH-positive fibers) neuronal loss as shown in Figure 4 and Supplementary Figure 4.

Microglia do not express nitric oxide synthase (iNOS) in its physiological state; it typically produces iNOS and releases a large amount of NO after stimulation by neurotoxin or after inflammatory injury. Consistent bursts of NO may be responsible for propagating neuronal death in several neurodegenerative diseases, including PD [30, 31]. Furthermore, several investigations have demonstrated that gene-knockouts of iNOS inhibitors oriNOS are able to attenuate DAN death in MPTP-induced PD mouse models [30, 31]. In our study, OYFCS significantly inhibited excessive NO production in LPS-stimulated microglia BV-2 cells as shown in Figure 1. Activated microglia produce increased levels of proinflammatory mediators such as TNF-*α*, IL-1*β*, and IL-6 which are neurotoxic. We observed that OYFCS was able to inhibit LPS-induced production of TNF-*α*, IL-1*β*, and IL-6 in BV-2 cells as shown in the Figure 2 and Supplementary Figure 1, showing that OYFCS might be beneficial for delaying the progression of inflammatory events seen in PD.

Activation of quiescent immune cells in the brain, such as microglial cells, is a hallmark of neuroinflammation. Subsequent production of proinflammatory mediators, such as TNF-*α*, IL-1*β*, and IL-6, is neurotoxic and either directly destroys DAN or leads to astrogliosis that encourages an influx of leucocytes from the periphery (predominantly T-lymphocytes) [10]. MPTP-induced PD mouse model demonstrates strong immune responses, including enhanced microglia activation, which suggests that MPTP mediates microglial overactivation, shown in Figure 3 and Supplementary Figure 2, which might contribute to the loss of neurons. In the study, while intraperitoneal injection of MPTP resulted in activation of glia-mediated inflammatory markers, such as Iba-1 and GFAP, pretreatment of OYF prevented this activation of glia cells and its mediators. Therefore, our data of* in vivo* and* in vitro* experiments suggest that OYF pretreatment was able to rescue mouse brains from undergoing DAN loss through inhibition of the proinflammatory mediators.

In view of more mechanisms needed elucidation, we used whole genome transcriptome analysis for OYF efficiency in the MPTP-induced PD model. In transcriptome analysis among control, MPTP-induced PD model, and OYF-pretreated PD (MPTP+OYF) mice, the most significant effects of OYF were on the genes involved in immunoregulatory and inflammatory pathways. Specifically, in OYF-treated mouse brains, 16 and 15 major signaling pathways were upregulated (Table 2) or downregulated (Table 3), respectively, and are either directly or indirectly related to immunoregulation. Therefore, the global transcriptional analysis provides overall insights into the mechanism of OYF and confirmed the neuroinflammation inhibition effects of the OYF in PD mouse model. The data might also provide clues for other neurodegenerative diseases.

## 5. Conclusions

In conclusion, our data showed that OYF can inhibit microglial activation and suppress secretion of proinflammatory cytokines, which collectively protects DAN from immune-mediate death. Motor deficits seen in the PD mouse model were prevented with OYF treatment. This study suggests that OYF can be used as an effective anti-inflammatory treatment in PD mouse model, providing a novel perspective for the treatment and prevention of PD patients, although more studies of clinical trials in PD might be necessary.

## Figures and Tables

**Table 1:** Primers applied in the experiments.

Gene	Primer (F/R)
TH	GTCTCAGAGCAGGATACCAAGC;
	CTCTCCTCGAATACCACAGCC
TNF-*α*	CCCTCACACTCAGATCATCTTCT;
	GCTACGACGTGGGCTACAG
IL-1*β*	GCAACTGTTCCTGAACTCAACT;
	ATCTTTTGGGGTCCGTCAACT
IL-6	TAGTCCTTCCTACCCCAATTTCC;
	TTGGTCCTTAGCCACTCCTTC
*β*-Actin	GGCTGTATTCCCCTCCATCG;
	CCAGTTGGTAACAATGCCATGT

**Table 2:** Profile 2 pathway enrichment.

	Pathway	DEGs genes with pathway annotation (105)	All genes with pathway annotation (7226)	P-value
1	Cytokine-cytokine receptor interaction	11 (12.79%)	264 (3.65%)	0.000270
2	Jak-STAT signaling pathway	8 (9.3%)	158 (2.19%)	0.000547
3	Complement and coagulation cascades	5 (5.81%)	88 (1.22%)	0.003837
4	Glycerolipid metabolism	4 (4.65%)	61 (0.84%)	0.005828
5	Galactose metabolism	3 (3.49%)	32 (0.44%)	0.006293
6	Pentose and glucuronate interconversions	3 (3.49%)	33 (0.46%)	0.006864
7	Cell adhesion molecules (CAMs)	6 (6.98%)	160 (2.21%)	0.011753
8	Steroid hormone biosynthesis	4 (4.65%)	85 (1.18%)	0.018248
9	Steroid biosynthesis	2 (2.33%)	19 (0.26%)	0.020996
10	Protein digestion and absorption	4 (4.65%)	90 (1.25%)	0.022026
11	VEGF signaling pathway	3 (3.49%)	58 (0.8%)	0.031408
12	alpha-Linolenic acid metabolism	2 (2.33%)	24 (0.33%)	0.032616
13	Ascorbate and aldarate metabolism	2 (2.33%)	26 (0.36%)	0.037825
14	Fc epsilon RI signaling pathway	3 (3.49%)	66 (0.91%)	0.043553
15	Leukocyte transendothelial migration	4 (4.65%)	114 (1.58%)	0.046522
16	Tight junction	5 (5.81%)	167 (2.31%)	0.048382
17	Thyroid hormone synthesis	3 (3.49%)	70 (0.97%)	0.050385
18	Adipocytokine signaling pathway	3 (3.49%)	71 (0.98%)	0.052170
19	Bile secretion	3 (3.49%)	71 (0.98%)	0.052170
20	Gastric acid secretion	3 (3.49%)	73 (1.01%)	0.055832

**Table 3:** Profile 5 pathway enrichment.

	Pathway	DEGs genes with pathway annotation (105)	All genes with pathway annotation (7226)	P-value
1	Retinol metabolism	8 (7.62%)	86 (1.19%)	0.000032
2	Cytokine-cytokine receptor interaction	12 (11.43%)	264 (3.65%)	0.000416
3	Complement and coagulation cascades	6 (5.71%)	88 (1.22%)	0.001686
4	Cell adhesion molecules (CAMs)	8 (7.62%)	160 (2.21%)	0.002193
5	Natural killer cell mediated cytotoxicity	7 (6.67%)	136 (1.88%)	0.003526
6	Phototransduction	3 (2.86%)	28 (0.39%)	0.007502
7	Hematopoietic cell lineage	5 (4.76%)	92 (1.27%)	0.010664
8	Drug metabolism-cytochrome P450	4 (3.81%)	64 (0.89%)	0.013728
9	Tyrosine metabolism	3 (2.86%)	36 (0.5%)	0.015038
10	Glycolysis/Gluconeogenesis	4 (3.81%)	69 (0.95%)	0.017682
11	Pancreatic secretion	5 (4.76%)	106 (1.47%)	0.018754
12	Fatty acid degradation	3 (2.86%)	47 (0.65%)	0.030463
13	Arachidonic acid metabolism	4 (3.81%)	83 (1.15%)	0.032257
14	Steroid hormone biosynthesis	4 (3.81%)	85 (1.18%)	0.034777
15	Phagosome	6 (5.71%)	170 (2.35%)	0.036951
16	Renin secretion	3 (2.86%)	69 (0.95%)	0.078539
17	Renin-angiotensin system	2 (1.9%)	33 (0.46%)	0.082549
18	Leukocyte transendothelial migration	4 (3.81%)	114 (1.58%)	0.083811
19	Salivary secretion	3 (2.86%)	77 (1.07%)	0.101104
20	Olfactory transduction	21 (20%)	1094 (15.14%)	0.106036

## Data Availability

The data used to support the findings of this study are available from the corresponding author upon request.

## Abbreviations

DA: Dopamine
DAN: Dopaminergic neurons/dopamine neurons
EAE: Experimental autoimmune encephalomyelitis
GFAP: Glial fibrillary acidic protein
Iba-1: Ionized calcium-binding adapter molecule 1
IL-1*β*: Interleukin 1 beta
IL-6: Interleukin 6
iNOS: Inducible nitric oxide synthase
LPS: Lipopolysaccharide
MPTP: 1-Methyl-4-phenyl-1,2,5,6-tetrahydropyridine
MS: Multiple sclerosis
NO: Nitrogen monoxide
OYF: Optimized Yinxieling Formula
OYFCS: OYF containing serum
PD: Parkinson's disease
PFA: Paraformaldehyde
SNpc: Substantia nigra pars compacta
TCM: Traditional Chinese medicine
TH: Tyrosine hydroxylase
TNF-*α*: Tumor necrosis factor-*α*.

## Supplementary Materials

Supplementary Materials Supplemental Figure 1: suppression of secretion of proinflammatory cytokines by OYFCS in LPS-stimulated BV-2 cells. Supplemental Figure 2: suppression of secretion of proinflammatory cytokines by OYF in PD mouse brains. Supplemental Figure 3: the glial activated in the PD mouse model and inhibited by the OYF pretreatment. Supplemental Figure 4: the protective effects of OYF against MPTP-mediated neuronal damage in the SNpc.
