# Supplementary material for: Curative Anti-Inflammatory Properties of Chinese Optimized Yinxieling Formula in Models of Parkinson's Disease
Source: Evid Based Complement Alternat Med. 2018 Nov 4;2018:6142065. doi: 10.1155/2018/6142065 (PMC6241364; doi:10.1155/2018/6142065)
Supplement: Supplementary Materials — Supplemental Figure 1: suppression of secretion of proinflammatory cytokines by OYFCS in LPS-stimulated BV-2 cells. Supplemental Figure 2: suppression of secretion of proinflammatory cytokines by OYF in PD mouse brains. Supplemental Figure 3: the glial activated in the PD mouse model and inhibited by the OYF pretreatment. Supplemental Figure 4: the protective effects of OYF against MPTP-mediated neuronal damage in the SNpc. [file 6142065.f1.pdf]

Supplement data

Supplementary method 1

Determination of TNF-α, IL-1β, and IL-6 by ELISA.

Measurement of cytokines (TNF-α, IL-1β, and IL-6) were determined by ELISA. BV-2 cells (1×10^5 cells per well in a 24 well plate) were exposed to different concentrations of OYFCS (2.5, 5 and 10%) for 2 h, followed by the addition of LPS (100ng/mL) or LPS alone in the presence of serum. The supernatants of the cultured BV-2 cells were collected 24 h after LPS stimulation; Midbrain tissue (100 mg) was rinsed with 1 × PBS, homogenized in 1 mL of 1 × PBS and stored overnight at -20°C. After two freeze-thaw cycles were performed to break the cell membranes, the homogenates were centrifuged for 5 minutes at 5000 g, 2 - 8°C. The supernatant was obtained and adjusted to the final protein concentration of 1 mg/mL for the cytokine testing. The concentrations of TNF-α, IL-1β, and IL-6 were measured by commercial ELISA Kit (CUSABIO, China) according to the manufacturer's protocol.

Legends for Supplemental Figures

Supplemental Figure 1

Suppression of secretion of pro-inflammatory cytokines by OYFCS in LPS-stimulated BV-2 cells.

Twenty-four hours prior to the LPS treatment (100ng/mL), BV2 cells were

subjected to a two-hour incubation with OYFCS at different concentrations. The protein level of TNF-α, IL-1β, and IL-6 were determined by ELISA. LPS exposure led to increased levels of pro-inflammatory TNF-α(A), IL-1β(B), and IL-6 (C). Pretreatment with OYFCS reduced levels of TNF-α, IL-1β and IL-6 significantly. Values are depicted as mean ± standard deviation (n=3) for three independent experiments. $^{\#\#}P< 0.01$ vs. control group and $^{*}P< 0.05$, $^{**}P< 0.01$ vs. LPS-treated group.

Supplemental Figure 2:

Suppression of secretion of pro-inflammatory cytokines by OYF in PD mouse brains

Midbrain have been obtained from mouse brains on day 7 after MPTP intoxication; then cytokine concentrations have been assessed using commercial ELISA kits. MPTP led to increased levels of pro-inflammatory TNF-α(A), IL-1β(B), and IL-6 (C). Pretreatment with OYF for 7 consecutive days significantly affect the levels of TNF-αand IL-1β. The elevated IL-6 level was only slightly reduced. All the values are depicted as mean ± standard deviation (A-C, n = 6 per group). $^{\#\#}P< 0.01$ vs. control group and $^{**}P< 0.01$ vs. MPTP-treated group.

Supplemental Figure 3

The glial activated in the PD mouse model and inhibited by the OYF pretreatment

The representative IHC staining for $GFAP^+$ astrocytes in SNpc. Scale bar = 50 or 25 μm.To obtain a better view of the GFAP-positive staining astroglia in SNpc, we zoomed in the figures as indicated in white rectangle.

Supplemental Figure 4:

The protective effects of OYF against MPTP-mediated neuronal damage in the SNpc

Representative images of SNpc TH-positive cell immunoreactivity (IR) with Scale bars, 200 or 100 μm. To obtain a better view of the TH-positive staining DAN cells in SNpc, we zoomed in the figures as indicated in white rectangle.

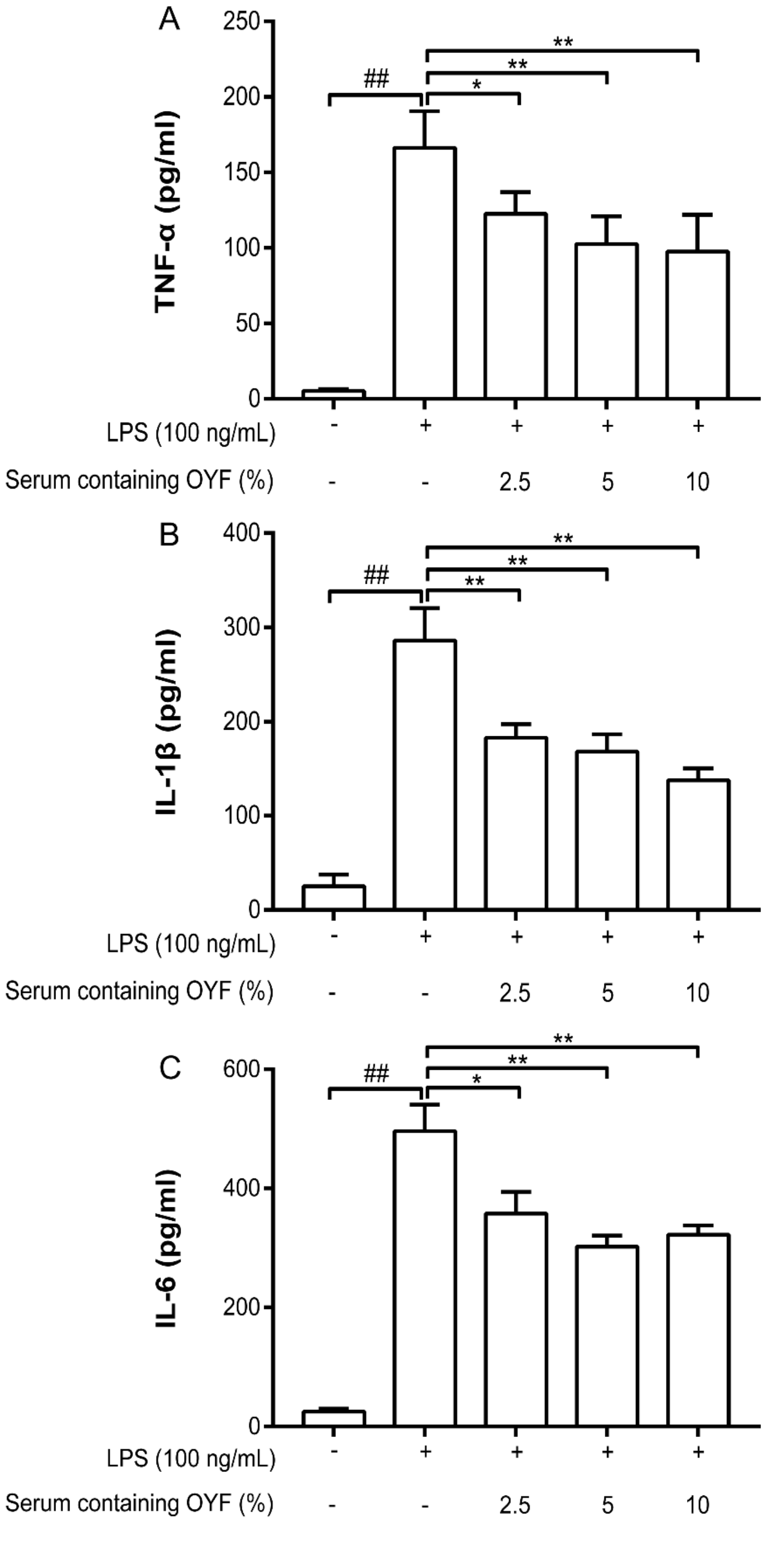

Supplemental Figure 1. Suppression of secretion of pro-inflammatory cytokines by OYFCS in LPS-stimulated BV-2 cells.

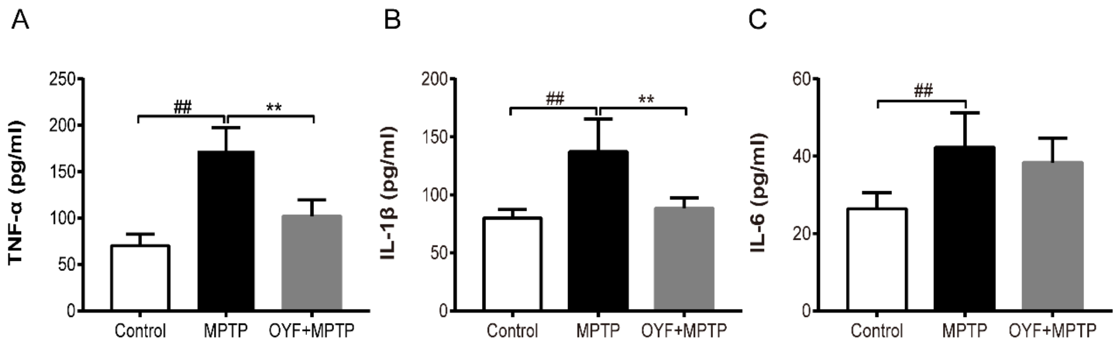

Supplemental Figure 2. Suppression of secretion of pro-inflammatory cytokines by OYF in PD mouse brains

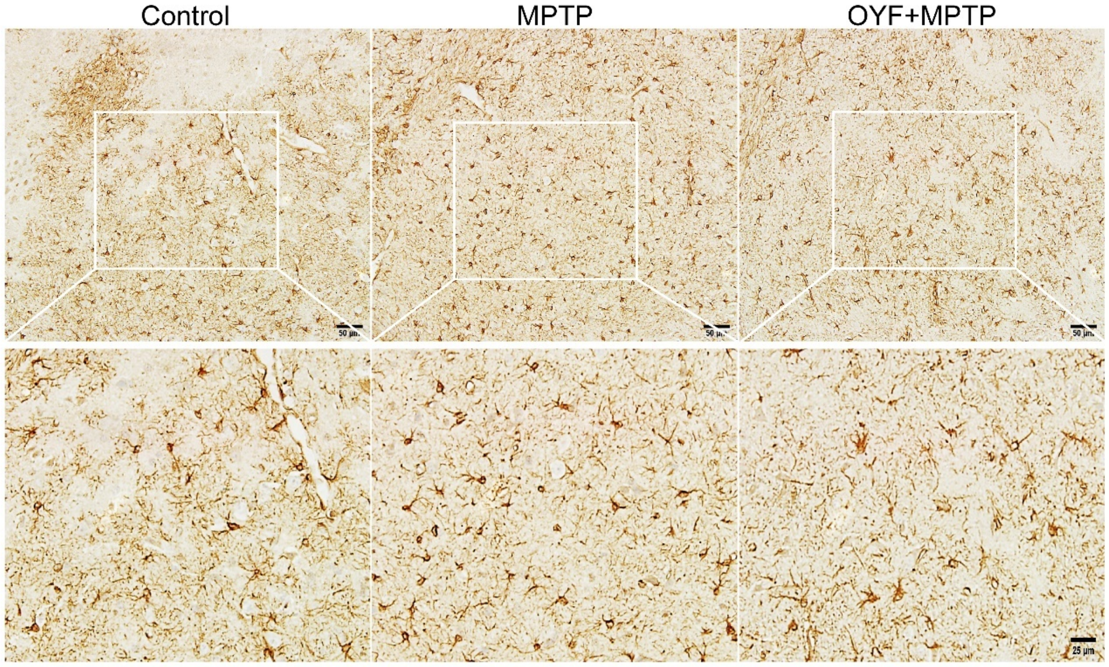

Supplemental Figure 3. The glial activated in the PD mouse model and inhibited by the OYF pretreatment

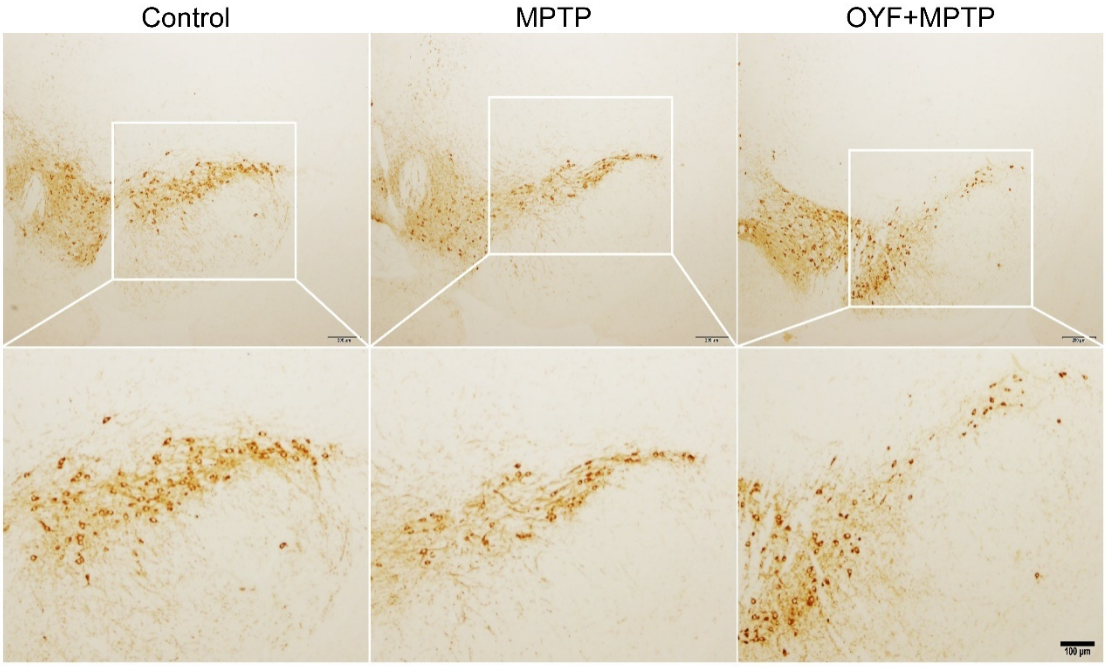

Supplemental Figure 4. The protective effects of OYF against MPTP-mediated neuronal damage in the SNpc
